# Supplementary material for: Differential compartmentalization of myeloid cell phenotypes and responses towards the CNS in Alzheimer’s disease
Source: Nat Commun. 2022 Nov 23;13:7210. doi: 10.1038/s41467-022-34719-2 (PMC9684147; doi:10.1038/s41467-022-34719-2)
Supplement: Supplementary file 1 — Supplementary Information [file 41467_2022_34719_MOESM1_ESM.pdf]

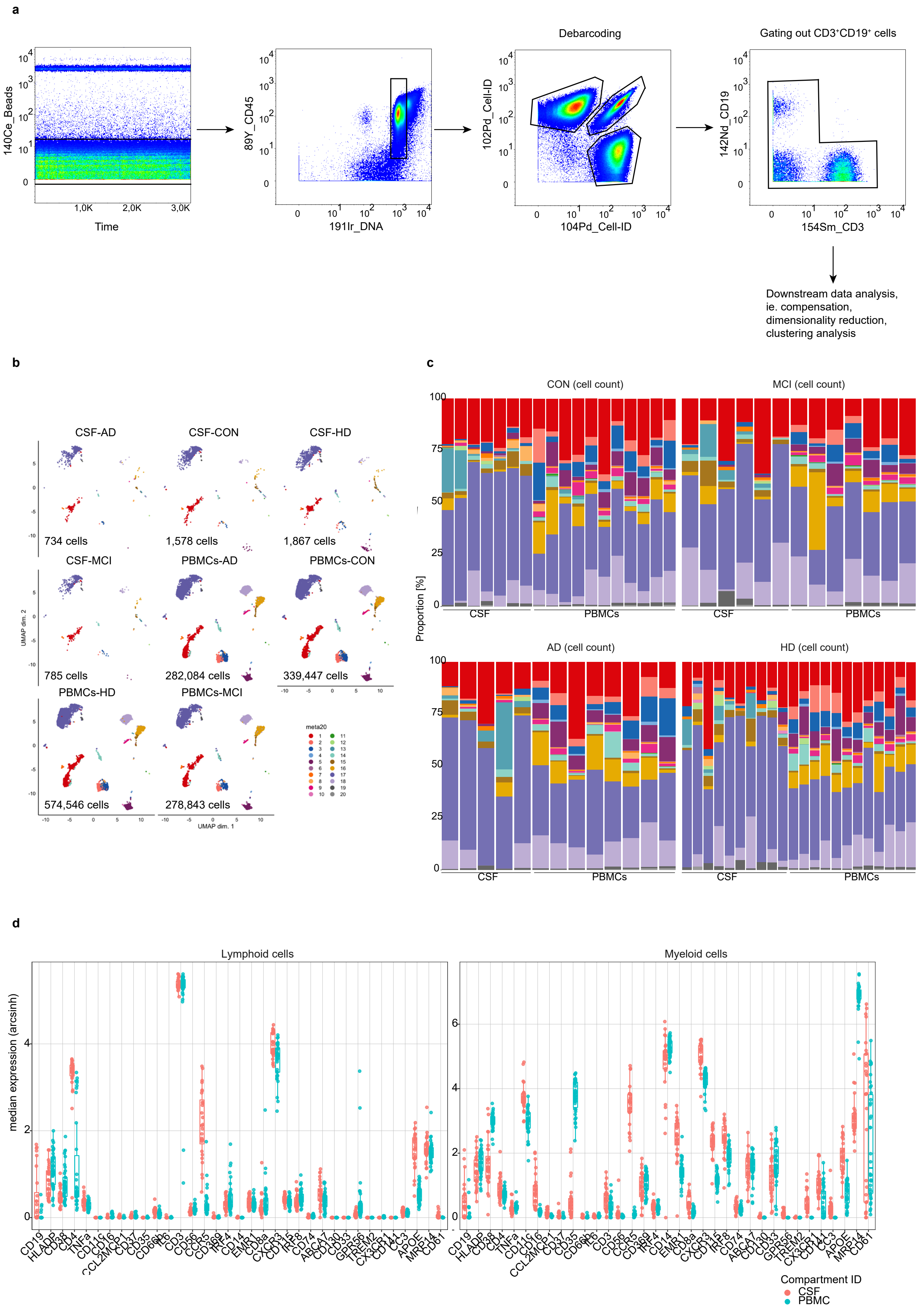

Supplementary Figure 1

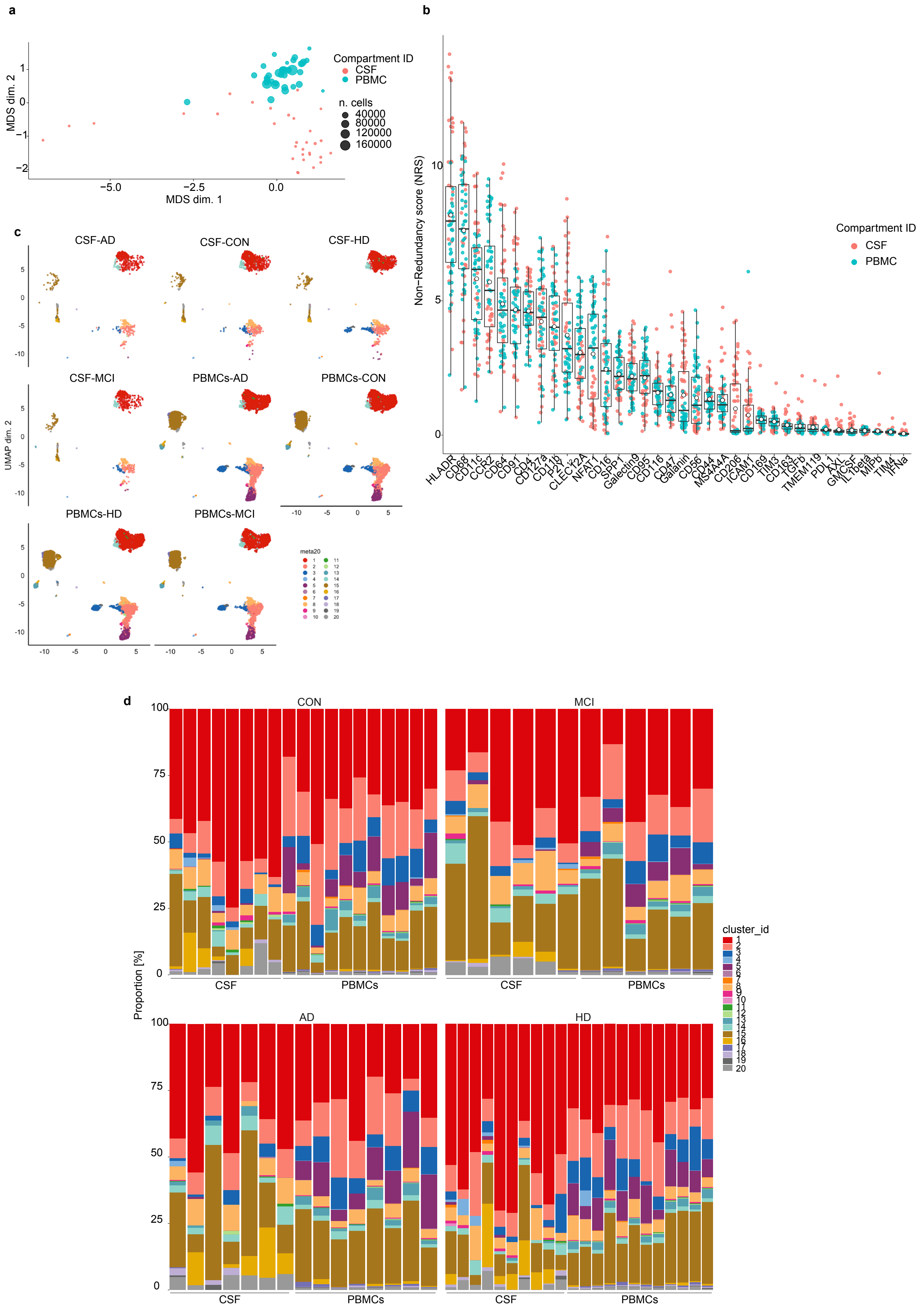

Supplementary Figure 2

A

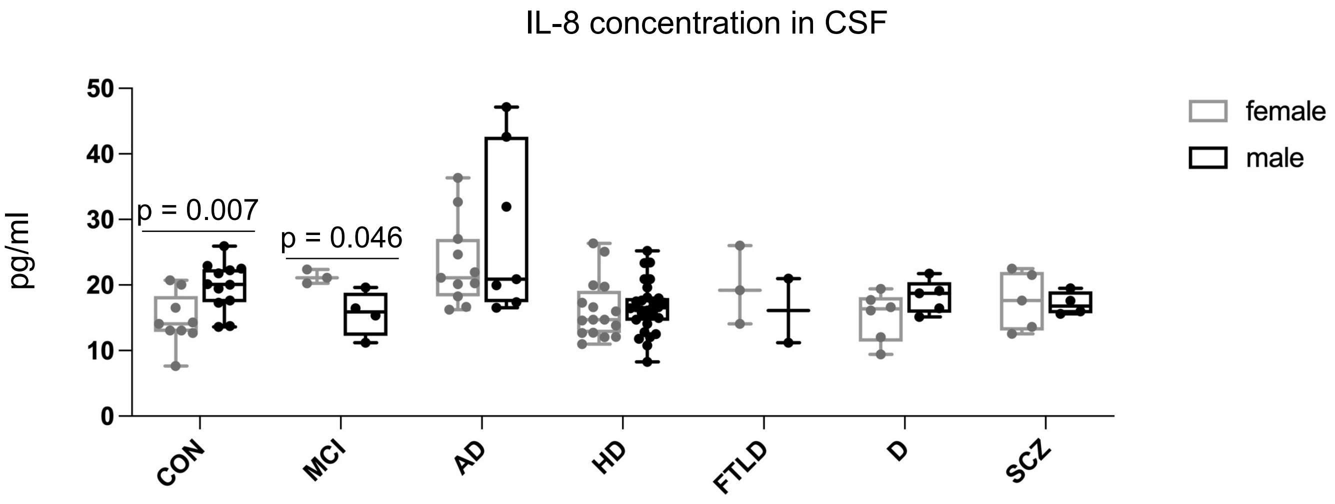

B

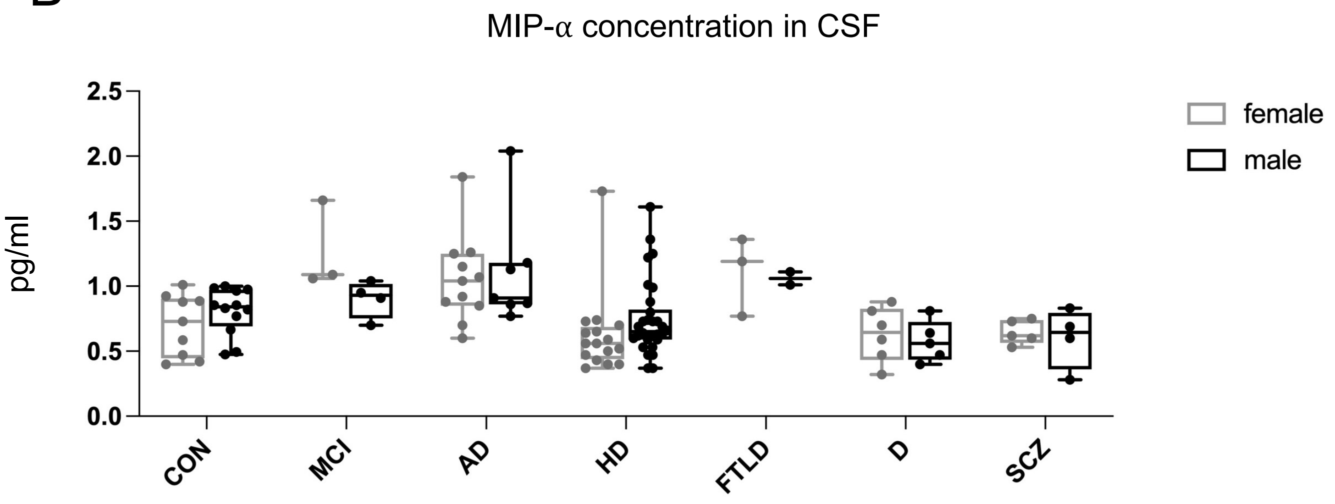

Supplementary Figure 3

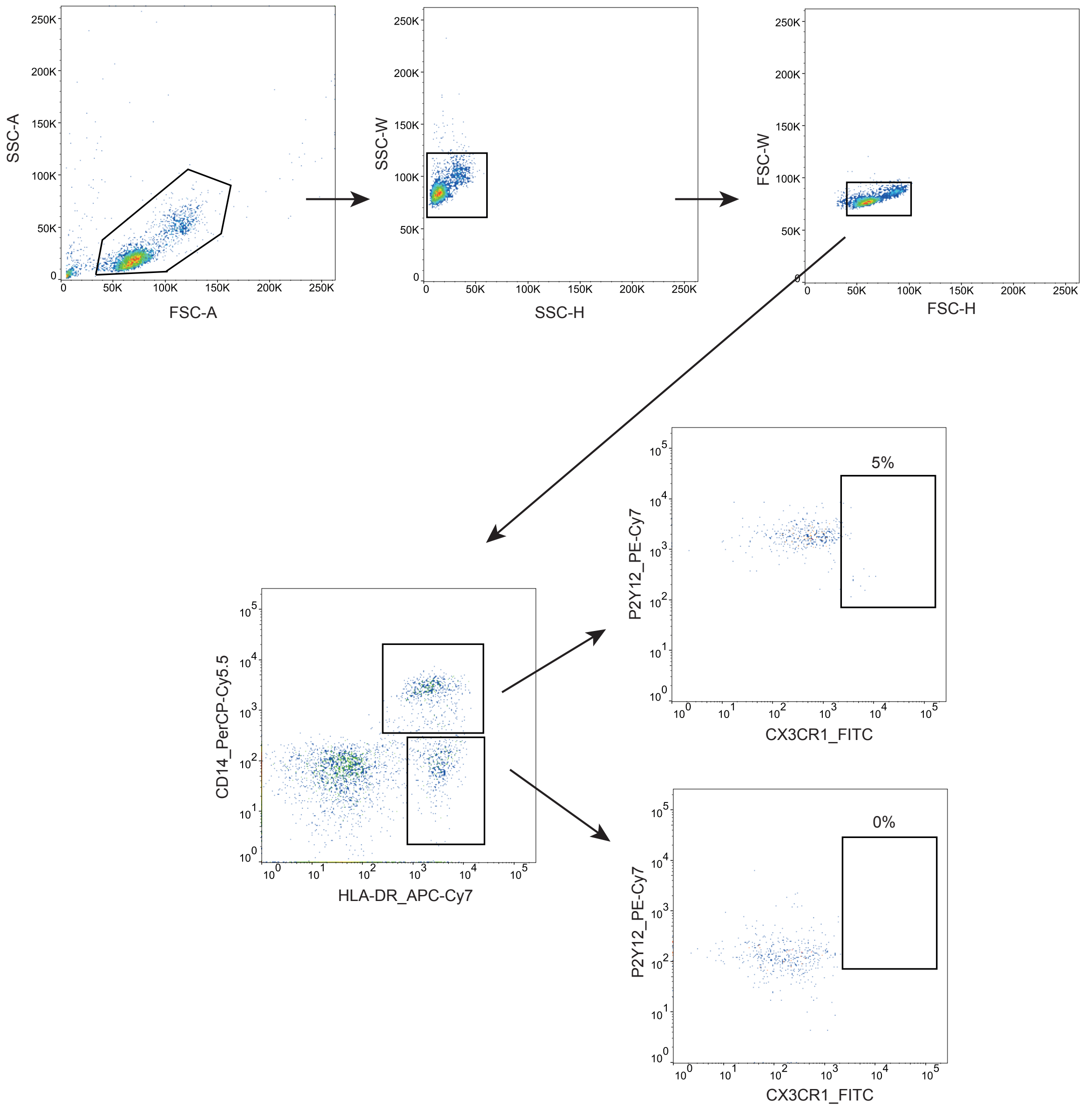

Supplementary Figure 4

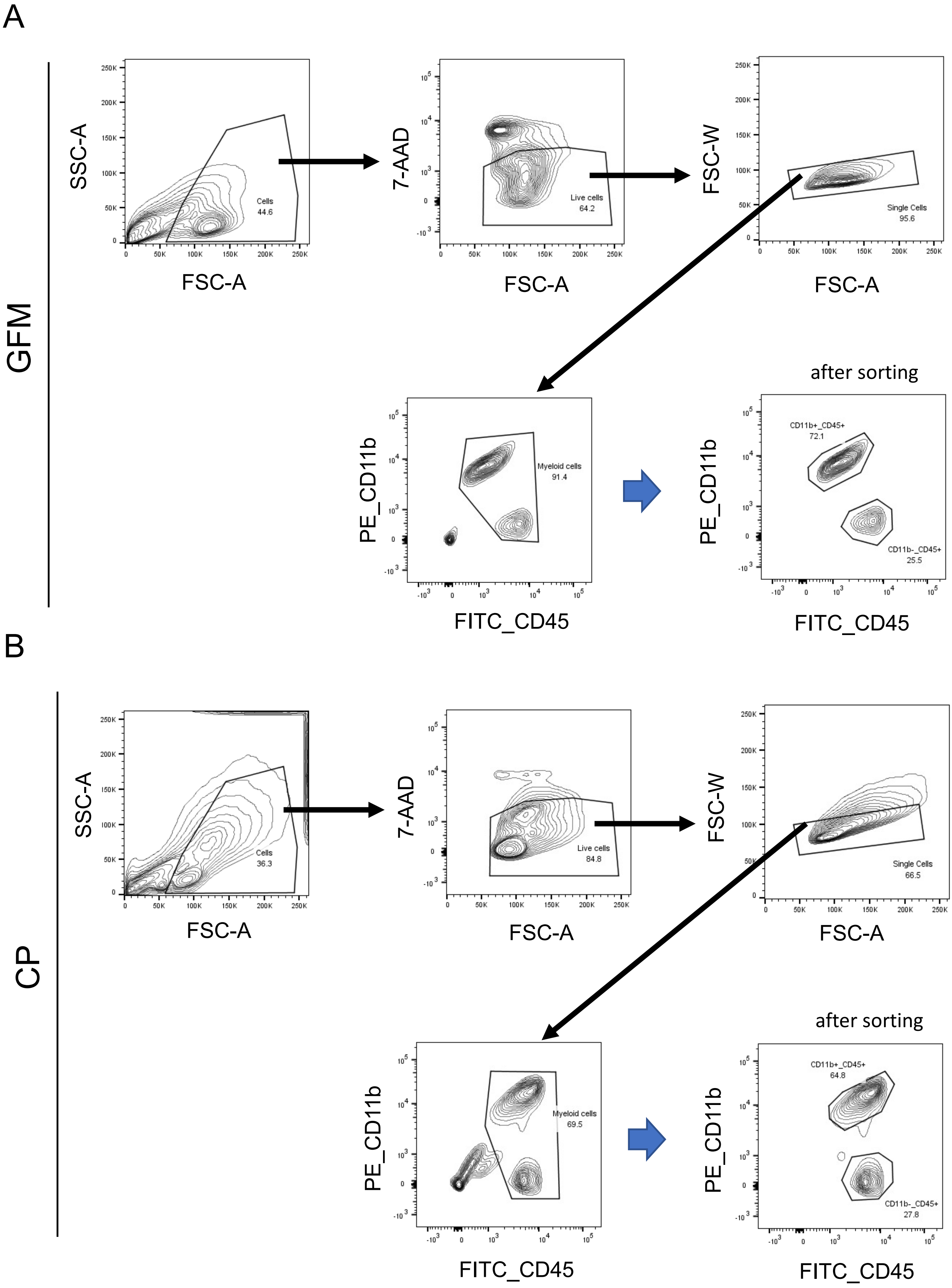

Suppl. Fig5_rev

Supplementary Figure 1 Gating strategy and cluster composition of PBMCs and CSF cells from *Panel 1*. (**A**) Gating strategy for CyTOF data prior to downstream analysis, selection of single $CD45^+$ cells, debarcoding based on boolean gating of palladium barcodes and exclusion of $CD3^+CD19^+$ double positives. (**B**) UMAP plots with all cells per group, color coded by cluster ID for the 1-20 clusters obtained with the FlowSOM algorithm. (**C**) Cluster abundance per sample. (**D**) Median marker expression (with arcsinh transformation) for all lymphoids and myeloids, showing overall differences between PBMCs (n = 38, biological replication) and CSF cells (n = 28, biological replication). Boxes extend from the 25th to 75th percentiles. Whisker plots show the min (smallest) and max (largest) values. The line in the box denotes the median. Each dot represents the value of each sample.

Supplementary Figure 2 Phenotypic characterization of PBMCs and CSF cells using Panel 2. (**A**) MDS plot from all cells from CSF (pink) and peripheral blood (green), each dot represents a sample and dot size represents number of cells per sample. (**B**) NRS for each marker and all samples (PBMCs: n = 38, biological replication and CSF cells: n = 28, biological replication). Each dot represents the per-sample NR scores (colored by compartment ID). The empty black circles indicate the mean NR scores from all the samples. Boxes extend from the 25th to 75th percentiles. Whisker plots show the min (smallest) and max (largest) values. The line in the box denotes the median. The top ten markers were selected as embedding parameters for clustering analysis. (**C**) UMAP plots with all cells per group, color coded by cluster ID for the 1-20 clusters obtained with the FlowSOM algorithm. (**D**) Cluster abundance per sample, color coded by cluster ID, compartment identities are indicated in the bottom.

Supplementary Figure 3 The scatter plots show gender-related differences in IL-8 (**A**) and MIP-1α (**B**) level in the CSF of CON individuals (9 females (F), 12 males (M), biological replication), or patients with MCI (3F, 4M), AD (11F, 7M), HD (16F, 30M), FTLD (3F, 2M), Depression (6F, 5M) or SCZ (5F, 4M). Boxes extend from the 25th to 75th percentiles. Whisker plots show the min (smallest) and max (largest) values. The line in the box denotes the median.

Each dot represents the value of each sample. Statistical significance was determined using unpaired, two-sided *t-test.*

Supplementary Figure 4 Gating strategy for FACS experiment in the **Figure 4E** and **4F**. $P2Y_{12}^{+}CXCR3^{+}$ cells were counted from the $HLADR^{+}CD14^{+}$ or $HLADR^{+}CD14^{-}$ populations as illustrated.

Supplementary Figure 5 Gating strategy for $CD45^{+}$ CNS cells used in **Figure 7** and **8.** All $CD45^{+}$ ($CD11b^{+}$ and $CD11b^{-}$) cells from GFM (**A**) and CP (**B**) were sorted using the same sorting strategy.

Supplementary Table 1. Donor and patients information

Related Figure	Group	CON	AD	MCI	HD	Depression	FTLD	SCZ
Fig. 1-6 (blood vs CSF)	**n**	21	18	7	46	11	5	9
	**Female/Male**	9/12	11/7	3/4	16/30	6/5	3/2	5/4
	**Age, mean +/- SD (range, years)**	62 +/- 14 (30-77)	72 +/- 10 (46-89)	78 +/- 1 (77-79)	49 +/- 11 (26-72)	57 +/- 19 (18-82)	62 +/- 4 (56-67)	46 +/- 19 (22-76)
	**CyTOF (n)**	11	8	7	12	-	-	-
	**Luminex (n)**	21	18	7	46	11	5	9
	**Tau (pg/ml), mean +/- SD (range)**	NA	597 +/- 453 (71-1584)	NA				
	**pTau (pg/ml), mean +/- SD (range)**		82,3 +/- 45,7 (29-167)					
	**Aß42 (pg/ml), mean +/- SD (range)**		662 +/- 168 (409-1002)					
	**Aß40 (pg/ml), mean +/- SD (range)**		15914 +/- 6228 (8421-27754)					
	**Aß42/40, mean +/- SD (range)**		0,5 +/- 0,3 (0,06-0,92)					
Fig. 7 and 8 (CP vs GFM)	**n**	7	6	NA				
	**Female/Male**	4/3	2/4					
	**Age, mean +/- SD (range, years)**	55-103 (84 +/- 16)	82-98 (84 +/- 12)					
	**CyTOF (n)**	7	6					
	**Luminex (n)**	-	-					
	**PMD, mean +/- SD (range, min)**	461 +/- 88 (330-590)	326 +/- 63 (215-385)					
	**CSF pH, mean +/- SD (range)**	6.7 +/- 0.2 (6.39-6.89)	6.5 +/- 0.4 (5.94-6.92)					
	**Braak Tau**	2.8 +/- 1.2 (1-4)	5.7 +/- 0.5 (5-6)					
	**Thal phase**	2.2 +/- 1.7 (0-4)	4.7 +/- 0.5 (4-5)					

Supplementary Table 2. The *Panel 1* -antibody list (CSF vs peripheral blood, **Fig. 1)**

target	Isotope tag	clone	company	Dilution
**surface**				
CD19	^{142}Nd	HIB19	Fluidigm	1:100
HLA-DP	^{143}Nd	Tü36	Biolegend	1:100
CD38	^{144}Nd	HIT2	Fluidigm	1:100
CD4	^{145}Nd	RPA-T4	Fluidigm	1:100
CD11c	^{147}Sm	Bu15	Fluidigm	1:100
CD16	^{148}Nd	3G8	Fluidigm	1:50
CD37	^{150}Nd	M-B371	Biolegend	1:100
CD35	^{151}Eu	594708	R&D	1:100
CD66b	^{152}Sm	80H3	Fluidigm	1:400
CD3	^{154}Sm	UCHT1	Fluidigm	1:200
CD56	^{155}Gd	NCAM16.2	Fluidigm	1:100
CCR5	^{156}Gd	NP6G4	Fluidigm	1:100
CD369	^{158}Gd	15E2	Biolegend	1:100
CD14	^{160}Dy	RM052	Fluidigm	1:200
EMR1	^{161}Dy	A10	Bio-Rad	1:100
CD8a	^{162}Dy	RPA-T8	Fluidigm	1:100
CXCR3	^{163}Dy	G025H7	Fluidigm	1:300
CD115	^{164}Dy	9-4D2-1E4	Biolegend	1:50
CD74	^{166}Er	LN2	Fluidigm	1:100
CD130	^{168}Er	2E1B02	Fluidigm	1:100
CD33	^{169}Tm	WM53	Fluidigm	1:100
GPR56	^{170}Er	CG4	Biolegend	1:50
TREM2	^{171}Yb	KBZ0318011	R&D	1:50
CX3CR1	^{172}Yb	2A9-1	Fluidigm	1:50
CD141	^{173}Yb	1A4	Fluidigm	1:100
CD61	^{Bi209}Di	VI-PL2	Fludigm	1:100
**intracellular**				
TNF	^{146}Nd	MAb11	Biolegend	1:100
CCL2	^{149}Sm	5D3-F7	Biolegend	1:200
IL6	^{153}Eu	MQ2-13A5	Biolegend	1:100
IRF4	^{159}Tb	IRF4.3E4	Biolegend	1:100
IRF8	^{165}Ho	7G11A45	Biolegend	1:100
ABCA7	^{167}Er	polyclonal	Merck	1:50
C3	^{174}Yb	4C6A8	Novus Biologicals	1:50
APOE	^{175}Lu	WUE-4	Novus Biologicals	1:100
MRP14-APC	-	MRP 1H9	Biolegend	1:200
APC	^{176}Yb	APC003	Fluidigm	1:100

Supplementary Table 3. The *Panel 2* -antibody list (CSF vs peripheral blood, **Fig. 2**)

target	Isotope tag	clone	company	Dilution
**surface**				
HLA-DR	^{141}Pr	L243	Biolegend	1:100
CD116	^{142}Nd	4H1	Fluidigm	1:100
CD11b	^{144}Nd	ICRF44	Fluidigm	1:100
CD4	^{145}Nd	RPA-T4	Fluidigm	1:100
CD64	^{146}Nd	10.1	Fluidigm	1:50
CD11c	^{147}Sm	3.9	Fluidigm	1:100
CD56	^{149}Sm	NCAM16.2	Fluidigm	1:100
CD95	^{152}Sm	DX2	Fluidigm	1:100
TIM3	^{153}Eu	F38-2E2	Miltenyi	1:100
CD172a	^{154}Sm	15-414	Biolegend	1:100
CD54 (ICAM1)	^{155}Gd	HA58	Biolegend	1:100
CD274 (PD-L1)	^{156}Gd	29E.2A3	Fluidigm	1:100
CD169	^{158}Gd	7-239	Fluidigm	1:100
CD163	^{160}Dy	GHI/61	Fluidigm	1:50
TIM4	^{161}Dy	9F4	Fluidigm	1:50
CD91	^{162}Dy	A2MR-α2	BD Bioscience	1:100
Galectin9	^{163}Dy	9M1-3	Fluidigm	1:100
TMEM119	^{164}Dy	A16075D	Biolegend	1:50
CD16	^{165}Ho	3G8	Fluidigm	1:50
CD44	^{166}Er	BJ18	Fluidigm	1:100
MS4A4A	^{167}Er	818112	R&D Systems	1:50
CD206	^{168}Er	15-2	Biolegend	1:50
TGF-β	^{169}Tm	TW4-2F8	Biolegend	1:50
P2Y12 (biotin)	-	polyclonal	Sigma-Aldrich	1:200
Biotin	^{170}Er	1D4-C5	Fluidigm	1:100
CCR2	^{171}Yb	K036C2	Biolegend	1:100
CLEC12A	^{173}Yb	50C1	Fluidigm	1:100
AXL	^{175}Lu	polyclonal	LSBio	1:50
CD47	$^{Bi209}Di$	CC2C6	Fluidigm	1:200
**intracellular**				
NFAT1	^{143}Nd	D43B1	Fluidigm	1:100
Galanin	^{148}Nd	581403	Fluidigm	1:100
MIP-1β (CCL4)	^{150}Nd	D211351	Fluidigm	1:200
CD68	^{151}Eu	Y1/82A	Biolegend	1:100
IL-1β	^{174}Yb	CRM56	eBioscience	1:100
OPN	^{172}Yb	polyclonal	LSBio	1:50
IFNα-APC	-	LT27:295	Miltenyi Biotec	1:100
APC	^{176}Yb	APC003	Fluidigm	1:100

Supplementary Table 4. The list of antibodies used for *ex vivo* experiment **(Fig. 5)**

target clone	Isotope tag	clone	company	dilution
**surface**				
CD45	89Y	HI30	Fluidigm	1:200
HLA-DR	141Pr	L243	Biolegend	1:100
CD19	142Nd	HIB19	Fluidigm	1:100
CD38	144Nd	HIT2	Fluidigm	1:100
CD4	145Nd	RPA-T4	Fluidigm	1:100
CD64	146Nd	10.1	Fluidigm	1:50
CD11c	147Sm	Bu15	Fluidigm	1:100
CD16	148Nd	3G8	Fluidigm	1:50
CD56	149Sm	NCAM16.2	Fluidigm	1:100
CD35	151Eu	594708	R&D	1:100
CD95	152Sm	DX2	Fluidigm	1:100
CD3	154Sm	UCHT1	Fluidigm	1:200
CCR5	156Gd	NP6G4	Fluidigm	1:100
CD14	160Gd	RM052	Fluidigm	1:200
CD36	161Dy	5-271	Biolegend	1:100
CD8	162Dy	RPA-T8	Fluidigm	1:100
CXCR3	163Dy	G025H7	Fluidigm	1:300
CD115	164Dy	9-4D2-1E4	Biolegend	1:50
P2Y12-biotin	-	polyclonal	Sigma-Aldrich	1:200
Biotin	165Ho	1D4-C5	Biolegend	1:100
CD141	166Er	M80	Fluidigm	1:100
CD33	169Tm	WM53	Fluidigm	1:100
GPR56	170Er	CG4	Biolegend	1:50
CCR2	171Yb	K036C2	Biolegend	1:100
Clec12A	173Yb	50C1	Fluidigm	1:100
CCR6	176Yb	11A9	Fluidigm	1:200
CD11b	209Bi	ICRF44	Fluidigm	1:200
**Intracellular**				
cPARP	143Nd	F21-852	Fluidigm	1:100
MIP-1β (CCL4)	150Nd	D211351	Fluidigm	1:200
CD68	153Eu	Y1/82A	Biolegend	1:100
IL-10	158Gd	JES3-9D7	Biolegend	1:100
IRF4	159Tb	IRF4.3E4	Biolegend	1:100
IRF8	167Er	7G11A45	Biolegend	1:100
IFNγ	168Er	B27	Fluidigm	1:100
IL-6	167Er	MQ2-13A5	Biolegend	1:100
OPN	172Yb	polyclonal	LSBio	1:50
IL-8-FITC	-	BH0814	Biolegend	1:200
FITC	174Yb	FIT-22	Fluidigm	1:100
TNF	175Lu	Mab11	Fluidigm	1:100

Supplementary Table 5. The list of antibodies used for *ex vivo* experiment **(Fig. 6)**

target clone	Isotope tag	clone	company	Dilution
**surface**				
CD45	89Y	HI30	Fluidigm	1:200
P2Y12-biotin	-	polyclonal	Sigma-Aldrich	1:200
Biotin	111Cd	1D4-C5	Biolegend	1.200
CD49d	141Pr	9F10	Fluidigm	1:100
CD19	142Nd	HIB19	Fluidigm	1:100
HLA-DR	143Nd	L243	Fluidigm	1.100
CD69	144Nd	FN50	Fluidigm	1:100
CD4	145Nd	RPA-T4	Fluidigm	1:100
CD64	146Nd	10.1	Fluidigm	1:50
CD123	147Sm	6H6	Biolegend	1:100
CD16	148Nd	3G8	Fluidigm	1:50
CD56	149Sm	NCAM16.2	Fluidigm	1:100
MERTK	151Eu	125518	R&D System	1:100
CD66b	152Sm	80H3	Fluidigm	1:400
CD3	154Sm	UCHT1	Fluidigm	1:200
CD11c	155Gd	44807	Fluidigm	1:100
CD61	156Gd	VI-PL2	Biolegend	1:100
CD1c	159Tb	L161	Biolegend	1:100
CD14	160Gd	RM052	Fluidigm	1:200
CD163	163Dy	GHI/61/Fluidigm	Fluidigm	1:50
CD115	164Dy	9-4D2-1E4	Biolegend	1:50
TGF-β (LAP)	166Er	TW4-2F8	Biolegend	1:50
CD206	168Er	15-2	Fluidigm	1:50
CCR2	169Tm	K036C2	Fluidigm	1:100
GPR56	170Er	CG4	Biolegend	1:50
TREM2	171Yb	237920	R&D System	1:50
CX3CR1	172Yb	2A9-1	Fluidigm	1:50
CXCR4	173Yb	12G5	Fluidigm	1:200
CCR6	176Yb	11A9	Fluidigm	1:200
CD11b	209Bi	ICRF44	Fluidigm	1:200
**Intracellular**				
CXCL12-FITC	-	79018	Thermofisher	1:50
FITC	114Cd	FIT-22	Biolegend	1:200
CXCL10-PE	-	4NY8UN	eBioscience™	1:50
PE	116Cd	PE001	Biolegend	1:200
MIP-1β (CCL4)	150Nd	D211351	Fluidigm	1:200
CD68	153Eu	Y1/82A	Biolegend	1:100
IL-10	158Gd	JES3-9D7	Biolegend	1:100
CTLA-4	161Dy	14D3	Fluidigm	1:100
TNF	162Dy	MAb11	Biolegend	1:100
IFNγ	165Ho	B27	Fluidigm	1:100
IL-6	167Er	MQ2-13A5	Biolegend	1:100
IL-1β	174Yb	CRM56	Invitrogen	1:100
ApoE	175Lu	WUE-4	Novus Biologicals	1:100

Supplementary Table 6. The list of antibodies used for an differential analysis of immune cells in CP and GFM - *Panel A* **(Fig. 7)**

target clone	Isotope tag	clone	company	dilution
**surface**				
CD19	^{142}Nd	HIB19	Fluidigm	1:100
HLA-DR	^{143}Nd	L243	Fluidigm	1:100
CD44	^{144}Nd	BJ18	Biolegend	1:50
CD4	^{145}Nd	RPA-T4	Fluidigm	1.100
CD11c	^{147}Sm	Bu15	Fluidigm	1:100
CD16	^{148}Nd	3G8	Fluidigm	1:50
CD86	^{150}Nd	IT2.2	Fluidigm	1:100
CD35	^{151}Eu	594708	R&D Systems	1:100
CD83	^{152}Sm	HB15e	Biolegend	1:100
CD3	^{154}Sm	UCHT1	Fluidigm	1:200
CD56	^{155}Gd	NCAM16.2	Fluidigm	1:100
CCR5	^{156}Gd	NP6G4	Fluidigm	1:100
CD169	^{158}Gd	7-239	Fluidigm	1:100
CD22	^{159}Tb	HIB22	Fluidigm	1:100
CD14	^{160}Dy	RMO52	Fluidigm	1:200
EMR1	^{161}Dy	A10	Bio-Rad	1:100
CD8a	^{162}Dy	RPA-T8	Fluidigm	1:100
CXCR3	^{163}Dy	G025H7	Fluidigm	1:300
CD115	^{164}Dy	9-4D2-1E4	Biolegend	1:50
CD74	^{166}Er	LN2	Fluidigm	1:100
CD130	^{168}Er	2E1B02	Fluidigm	1:100
TGF-β	^{169}Tm	TW4-2F8	Biolegend	1:50
GPR56	^{170}Er	CG4	Biolegend	1.50
CXCR5	^{171}Yb	RF8B2	Fluidigm	1.100
CX3CR1	^{172}Yb	2A9-1	Biolegend	1.50
CD141	^{173}Yb	1A4	Fluidigm	1:100
AXL	^{175}Lu	polyclonal	LSBio	1:50
CD61	$^{Bi209}Di$	VI-PL2	Fluidigm	1:100
**intracellular**				
TNF	^{146}Nd	MAb11	Fluidigm	1:100
CCL2	^{149}Sm	5D3-F7	Biolegend	1:200
IL-6	^{153}Eu	MQ2-13A5	Biolegend	1:100
IRF8	^{165}Ho	7G11A45	Biolegend	1:100
ABCA7	^{167}Er	polyclonal	Merck	1:50
C3	^{174}Yb	4C6A8	Novus Biologicals	1:50
MRP14-APC	-	MRP 1H9	Biolegend	1:200
APC	^{176}Yb	APC003	Fluidigm	1:100

Supplementary Table 7. The list of antibodies used for an differential analysis of immune cells in CP and GFM - *Panel B* **(Fig. 8)**

target clone	Isotope tag	clone	company	dilution
**surface**				
HLA-ABC	^{141}Pr	W6/36	Fluidigm	1:200
CD116	^{142}Nd	4H1	Fluidigm	1:100
CD11b	^{144}Nd	ICRF44	Fluidigm	1:100
CD18	^{145}Nd	TS1/18	Biolegend	1:100
CD64	^{146}Nd	10.1	Fluidigm	1:50
CD11c	^{147}Sm	3.9	Fluidigm	1:100
CD14	^{148}Nd	RM052	Fluidigm	1:100
CD95	^{152}Sm	DX2	Fluidigm	1:100
TIM3	^{153}Eu	F38-2E2	Miltenyi Biotec	1:100
CD172a	^{154}Sm	15-414	Biolegend	1:100
CD54	^{155}Gd	HA58	Biolegend	1:100
PDL1	^{156}Gd	29E.2A3	Fluidigm	1:100
CD369	^{158}Gd	15E2	Biolegend	1:100
CD163	^{160}Dy	GHI/61	Fluidigm	1:100
CD36	^{161}Dy	5-271	Biolegend	1:100
CD91	^{162}Dy	A2MR-α2	BD Bioscience	1:100
GLUT 5	^{163}Dy	195205	R&D Systems	1:100
TMEM119	^{164}Dy	A16075D	Biolegend	1:50
CD32-PE	-	6C4(CD32)	eBioscience	1:100
PE	^{165}Ho	PE001	Fluidigm	1:100
MS4A4A	^{167}Er	818112	R&D Systems	1:50
CD206	^{168}Er	15-2	Biolegend	1:50
CD33	^{169}Tm	WM53	Fluidigm	1:100
P2Y12 (biotin)	-	polyclonal	Sigma-Aldrich	1:200
Biotin	^{170}Er	1D4-C5	Fluidigm	1:100
TREM2	^{171}Yb	KBZ0318011	R&D	1:50
CD9	^{172}Yb	SN4 C3-3A2	Fluidigm	1:100
CLEC12A	^{173}Yb	50C1	Fluidigm	1:100
CD47	^{Bi209}Di	CC2C6	Fluidigm	1:200
**intracellular**				
IKZF1	^{143}Nd	16B5C71	Fluidigm	1:100
MIP-1β (CCL4)	^{150}Nd	D211351	Fluidigm	1:200
CD68	^{151}Eu	Y1/82A	Biolegend	1:100
GMCSF	^{159}Tb	BVD2-21C11	Biolegend	1:100
IL-10	^{166}Er	JES3-9D7	Biolegend	1:100
IL-1β	^{174}Yb	CRM56	eBioscience	1:100
APOe	^{175}Lu	polyclonal	LSBio	1:100
IFNα-APC	-	LT27:295	Miltenyi Biotec	1:100
APC	^{176}Yb	APC003	Fluidigm	1:100
