# Supplementary material for: Differential compartmentalization of myeloid cell phenotypes and responses towards the CNS in Alzheimer’s disease
Source: Nat Commun. 2022 Nov 23;13:7210. doi: 10.1038/s41467-022-34719-2 (PMC9684147; doi:10.1038/s41467-022-34719-2)
Supplement: Supplementary file 3 — Reporting Summary [file 41467_2022_34719_MOESM3_ESM.pdf]

nature portfolio

Corresponding author(s): Chotima Böttcher

Last updated by author(s): Oct 26, 2022

Reporting Summary

Nature Portfolio wishes to improve the reproducibility of the work that we publish. This form provides structure for consistency and transparency in reporting. For further information on Nature Portfolio policies, see our Editorial Policies and the Editorial Policy Checklist.

Statistics

For all statistical analyses, confirm that the following items are present in the figure legend, table legend, main text, or Methods section.

n/a	Confirmed	
☐	☒	The exact sample size (n) for each experimental group/condition, given as a discrete number and unit of measurement
☐	☒	A statement on whether measurements were taken from distinct samples or whether the same sample was measured repeatedly
☐	☒	The statistical test(s) used AND whether they are one- or two-sided<br>*Only common tests should be described solely by name; describe more complex techniques in the Methods section.*
☐	☒	A description of all covariates tested
☐	☒	A description of any assumptions or corrections, such as tests of normality and adjustment for multiple comparisons
☐	☒	A full description of the statistical parameters including central tendency (e.g. means) or other basic estimates (e.g. regression coefficient) AND variation (e.g. standard deviation) or associated estimates of uncertainty (e.g. confidence intervals)
☐	☒	For null hypothesis testing, the test statistic (e.g. F, t, r) with confidence intervals, effect sizes, degrees of freedom and P value noted<br>*Give P values as exact values whenever suitable.*
☒	☐	For Bayesian analysis, information on the choice of priors and Markov chain Monte Carlo settings
☐	☒	For hierarchical and complex designs, identification of the appropriate level for tests and full reporting of outcomes
☐	☒	Estimates of effect sizes (e.g. Cohen's d, Pearson's r), indicating how they were calculated

Our web collection on statistics for biologists contains articles on many of the points above.

Software and code

Policy information about availability of computer code

Data collection	CyTOF acquisition software 6.5.236 and flow cytometry acquisition: BD FACSDiva Software 6.1.3
Data analysis	FlowJo software 10.4.2; Cytobank (www.cytobank.org); R/Bioconductor packages: CATALYST (see. Methods page 32,33)

For manuscripts utilizing custom algorithms or software that are central to the research but not yet described in published literature, software must be made available to editors and reviewers. We strongly encourage code deposition in a community repository (e.g. GitHub). See the Nature Portfolio guidelines for submitting code & software for further information.

Data

Policy information about availability of data

All manuscripts must include a data availability statement. This statement should provide the following information, where applicable:

- Accession codes, unique identifiers, or web links for publicly available datasets
- A description of any restrictions on data availability
- For clinical datasets or third party data, please ensure that the statement adheres to our policy

Source data associated with Fig. 1,2,5,6,7 & 8 will be available at https://flowrepository.org/id/FR-FCM-Z5XD

Field-specific reporting

Life sciences study design

All studies must disclose on these points even when the disclosure is negative.

Sample size	No priori statistical methods were used to predetermine sample sizes due to sample accessibility and insufficient previous data to enable this. However, sample sizes in this study are similar to our previous studies (Böttcher et al. 2019; Böttcher et al. 2020; Sankowski et al. 2019; Masuda et al. 2019, etc.) in which significant differences between conditions could be observed.
Data exclusions	The filtering parameters for differential analysis of cell population abundance was set to minimum number of cells = 3 in at least minimum number of samples = number of samples in each group. Only samples with more than 50 cells were considered for clustering analysis.
Replication	For CyTOF measurement, the marker expression of each individual samples was measured twice (antibody panel 1/A and 2/B) and were reliably reproduced. For in vitro experiments (i.e. Seahorse, 13C-glucose tracing and in vitro stimulation), the experiments were acquired at least twice to ensure the reproducibility.
Randomization	No method of randomization was used in this study. However, samples were acquired in different batches ensuring that all conditions analysed were determined in parallel with the AD samples.
Blinding	No specific blinding was necessary, since we performed data processing and data analysis by algorithms in an unsupervised manner and thus excluded the possibility of biased results.

Reporting for specific materials, systems and methods

We require information from authors about some types of materials, experimental systems and methods used in many studies. Here, indicate whether each material, system or method listed is relevant to your study. If you are not sure if a list item applies to your research, read the appropriate section before selecting a response.

Materials & experimental systems

n/a	Involved in the study	
☐	☒	Antibodies
☒	☐	Eukaryotic cell lines
☒	☐	Palaeontology and archaeology
☒	☐	Animals and other organisms
☐	☒	Human research participants
☒	☐	Clinical data
☒	☐	Dual use research of concern

Methods

n/a	Involved in the study	
☒	☐	ChIP-seq
☐	☒	Flow cytometry
☒	☐	MRI-based neuroimaging

Antibodies

Antibodies used	HLA-DR (clone L243, cat# 307651) Biolegend, CD116 (clone 4H1, cat# 305902) Fluidigm, NFAT1 (clone D43B1, cat# 3143023A) Fluidigm, CD11b (clone ICRF44, cat# 3209003B) Fluidigm, CD4 (clone RPA-T4, cat# 3145001B) Fluidigm, CD64 (clone 10.1, cat# 3146006B) Fluidigm, CD11c (clone 3.9, cat# 3146014B) Fluidigm, Galanin (clone 581403, cat# 3148016B) Fluidigm, CD56 (clone NCAM16.2, cat# 3149021B) Fluidigm, MIP-1β/CCL4 (clone D211351, cat# 3150004B) Fluidigm, CD68 (clone Y1/82A, cat# 333802) Biolegend, CD95 (clone DX2, 3152017B) Fluidigm, TIM3 (clone F38-2E2, cat# 3153008B) Miltenyi, CD172a (clone 15-414, cat# 372102) Biolegend, CD54/ICAM1 (clone HA58, cat#353102) Biolegend, CD274/PD-L1 (clone 29E.2A3, cat# 3156026B) Fluidigm, CD169 (clone 7-239, cat# 3158027B) Fluidigm, CD163 (clone GHI/61, cat# 333602) Fluidigm, TIM4 (clone 9F4, cat# 3161021B) Fluidigm, CD91 (clone A2MR-2, cat# 550495) BD Bioscience, Galectin9 (clone 9M1-3, cat# 3163002B) Fluidigm, TMEM119 (clone A16075D, cat# 853302) Biolegend, CD16 (clone 3G8, cat# 3165001B) Fluidigm, CD44 (clone BJ18, cat# 3166001B) Fluidigm, MS4A4A (clone 818112, cat# MAB7797) R&D Systems, CD206 (clone 15-2, cat# 3168008B) Biolegend, TGF-β (clone TW4-2F8, cat# 349602) Biolegend, P2Y12-biotin (polyclonal, cat# HPA014518) Sigma-Aldrich, Biotin (clone 1D4-C5, cat# 3143008B) Fluidigm, CCR2 (clone K036C2, cat# 3153023B) Biolegend, OPN (polyclonal, cat# C99283) LSBio, CLEC12A (clone 50C1, cat# 3173007B) Fluidigm, IL-1β (clone CRM56, cat# 14-7018-85) eBioscience, AXL (ploytclonal, cat# C100246) LSBio, IFN-APC (clone LT27:295, cat# 130-092-602) Miltenyi Biotec, APC (Clone APC003, cat# 3176007B) Fluidigm, CD47 (clone CC2C6, cat# 3209004B) Fluidigm, CD19 (clone HIB19, cat# 3142001B) Fluidigm, HLA-DP (clone Tü36, cat# 361602) Biolegend, CD38 (clone HIT2, cat# 3167001B) Fluidigm, TNF (clone MAb11, cat# 3175023B) DVS Sciences, CD11c (clone Bu15, cat# 3147008B) Fluidigm, CCL2 (clone 5D3-F7, cat# 502602) Biolegend, CD37 (clone M-B371, cat# 356302) Biolegend, CD35 (clone 594708, cat# MAB5748) R&D, CD66b (clone 80H3, cat# 3152011B) Fluidigm, IL-6 (clone MQ2-13A5, cat# 501115) Biolegend, CD3 (clone UCHT1, cat# 3154003B) Fluidigm, CCR5 (clone NP6G4, cat# 3171017A) Fluidigm, CD369 (clone 15E2, cat# 355402) Biolegend, IRF4 (clone IRF4.3E4, cat# 646402) Biolegend, CD14 (clone RM052, cat# 3160006B) Fluidigm, EMR1 (clone A10, cat# MCA2674GA) Biorad, CD8a (clone RPA-T8, cat# 3162015B) Fludigim, CXCR3 (clone G025H7, cat# 3163004B) Fluidigm, CD115 (clone 9-4D2-1E4, cat# 347302) Biolegend, IRF8 (clone 7G11A45, cat# 656502) Biolegend, CD74 (clone LN2, cat# 3166018B) Fluidigm, ABCA7 (polyclonal, cat# ABC349) Merck, CD130 (clone 2E1B02, cat# 3168016B) Fluidigm, CD33 (clone WM53, cat# 3169010B) Fluidigm, GPR56 (clone CG4, cat# 358202) Biolegend, TREM2 (clone KBZ0318011, cat# MAB17291) R&D, CX3CR1 (clone 2A9-1, cat# 3172017B) Fluidigm, CD141 (clone 1A4, cat# 3173002B) Fluidigm, C3 (clone 4C6A8, cat# NBP2-61855) Novus Biologicals, APOE (clone WUE-4, cat# NB110-60531) Novus Biologicals, MRP14 (S100A9)-APC (clone MRP 1H9, cat# 350708) Biolegend, CD61 (clone VI-PL2, cat# 336402) Biolegend, CD45 (clone HI30, cat# 3089003B) Fluidigm, cPARP (clone

F21-852, cat# 3143011A) Fluidigm, IL-10 (clone JES3-9D7, cat# 501423) Biolegend, IL8-PE (clone E8N1, cat# 511408) Biolegend, IRF8 (clone 7G11A45, cat# 656502) Biolegend, IFNγ (clone B27, cat#3168005B) Fluidigm, MIPα-APC (clone CCL3, cat# 130-103-630), CXCL12-FITC (clone 79018, cat# MA5-23547) Thermofisher, FITC (clone FIT-22, cat# 408304) Biolegend, CXCL10-PE (clone 4NY8UN, cat# 12-9744-42) eBioscience, PE (clone PE001, cat# 408105) Biolegend, CD49d (clone 9F10, cat# 3141004B) Fluidigm, CD69 (clone FN50, cat# 3144018B) Fluidigm, CD123 (clone 6H6, cat# 306002) Biolegend, MERTK (clone 125518, cat# MAB8912) R&D, CD1c (clone L161, cat# 331502) Biolegend, CTLA-4 (clone 14D3, cat# 3161004B) Fluidigm, CXCR4 (clone 12G5, cat# 3173001B) Fluidigm, CCR6 (clone G034E3, cat# 3176022A) Fluidigm, CD86 (clone IT2.2, cat# 3156008B) Fluidigm, CD83 (clone HB15e, cat# 305302) Biolegend, CXCR5 (clone RF8B2, cat# 3171014B) Fluidigm, HLA-ABC (clone W6/36, cat# 3141010B) Fluidigm, IKZF1 (clone 16B5C71, cat# 3143024B) Fluidigm, CD18 (clone TS1/18, cat# 302102) Biolegend, GM-CSF (clone BVD2-21C11, cat# 3159008B) DVS Science, CD36 (clone 5-271, cat# 336215) Biolegend, GLUT-5 (clone 195205, cat# MAB1349) R&D, CD32-PE (clone 6C4(CD32), cat# 11-0329-41) eBioscience, CD9 (clone SN4 C3-3A2, cat# 3172010B) Fluidigm, CD14-PerCP/Cy5.5 (clone HCD14, cat# 325622) Biolegend, HLA-DR-APC/Cy7 (clone L243, cat# 307618) Biolegend, CXCR1-FITC (clone 2A9-1, cat# 341606) Biolegend, TNF-Brillant Violet 421 (clone Mab11, cat# 502932) Biolegend.

Validation	All antibodies were validated for use in the human immune cells including brain microglia using flow cytometry and subsequently mass cytometry.

Human research participants

Policy information about studies involving human research participants

Population characteristics	see Supplementary Table 1
Recruitment	Patients and brain donors were prospectively recruited. There was no inclusion/exclusion criteria for medication therapy and/or other confounders, and thus we could not exclude impacts of any confounders.<br>See also Supplementary Table 1 and Online Methods (page 30, 31)
Ethics oversight	The study was registered and approved by the Ethics Commission of Charité–Universitätsmedizin Berlin (Ethikkommission der Charité–Universitätsmedizin Berlin; registration number EA1/386/20), Berlin, Germany.<br>Human brain tissue was obtained through the Netherlands Brain Bank (www.brainbank.nl). The Netherlands Brain Bank received permission to perform autopsies and to use tissue and medical records from the Ethical Committee of the VU University medical center (VUmc, Amsterdam, The Netherlands).

Note that full information on the approval of the study protocol must also be provided in the manuscript.

Flow Cytometry

Plots

Confirm that:

- [x] The axis labels state the marker and fluorochrome used (e.g. CD4-FITC).
- [x] The axis scales are clearly visible. Include numbers along axes only for bottom left plot of group (a 'group' is an analysis of identical markers).
- [x] All plots are contour plots with outliers or pseudocolor plots.
- [x] A numerical value for number of cells or percentage (with statistics) is provided.

Methodology

Sample preparation	For PBMCs:<br>PBMCs were isolated from EDTA-blood through Biocoll (Biochrom GmbH, Berlin, Germany) density centrifugation at 1,200 x g for 20 minutes at room temperature. The blood mononuclear cell fraction was recovered and washed twice in phosphate-buffered saline (PBS; Biochrom GmbH) at 300 x g for 10 minutes. The cell pellet was then fixed with fixation/stabilization buffer (SmartTube) and frozen at –80 °C until analysis by mass cytometry.<br><br>For CSF cells: CSF was centrifuged once at 300 x g for 10 minutes. The cell pellet was then fixed with fixation/stabilization buffer (SmartTube) and frozen at –80 °C until analysis by mass cytometry.<br><br>For brain immune cells:<br>Brain cell suspension was separated though Percoll (Amersham, GE Healthcare) at 3,220 x g for 30 minutes (4°C). The middle layer was carefully taken out without disturbing the myelin layer and washed, followed by cell sorting using flow cytometry. CD45+ cells were sorted and was then fixed with fixation/stabilization buffer (SmartTube) and frozen at –80 °C until analysis by mass cytometry.<br><br>See also Online Method (page 30, 31)
Instrument	FACSAriaIII and CantoII<br>see Online Method (page 31, 34)
Software	Data collection: BD FACSDiva Software 6.1.3<br>Data analysis: FlowJo software 10.4.2

Cell population abundance	More than 60% of single cells were 7-AAD negative. More than 90% of live single cells were CD45-positive.<br>see Online Method (page 31) and Supplementary Figure 5
Gating strategy	Cell duplets were excluded and (for cell sorting) 7-AAD-negative live cells were selected. CD45+ Cells (both CD11bhi and CD11blow) were FACS-sorted.<br><br>See Supplementary Figure 4 and 5.

☒ Tick this box to confirm that a figure exemplifying the gating strategy is provided in the Supplementary Information.
